# Tumor derived exosome induction of the CD3+CD8+ T cell tolerogenic phenotype

**DOI:** 10.1186/2051-1426-1-S1-P165

**Published:** 2013-11-07

**Authors:** Brian Maybruck, Brian Gastman

**Affiliations:** 1 Immunology, Cleveland Clinic, Cleveland, OH, USA

## Background

Traditionally, CD8+ T cells have been defined as being immunogenic; however, they have also been shown to demonstrate a tolerogenic phenotype. One area less studied, regarding the tolerogenic phenotype of CD8+ T cells, is their role in cancer. What has been described is that their suppressive phenotype is suggested to be developed in the tumor microenvironment and can be correlated with the CD8+ T cells becoming CD27/28-. What is unclear is the general mechanism that may trigger the tolerogenic development in CD8+ T cells. Via multiple cancer cell lines, this research identified that tumor derived (TD) exosomes will cause the development of the tolerogenic phenotype in CD3+CD8+ T cells.

## Methods

Conditioned growth media from tumor cell lines were processed by an exosome precipitation solution to purify exosomes. Confirmation of exosome isolation was based upon the identification of 10 commonly found exosomal proteins from the purification. Human CD3+CD8+ T cells were incubated with TD exosomes, tumor conditioned growth medium with and without exosomes, and exosome-free complete RPMI. Following this incubation, the CD3+CD8+ T cells were analyzed for CD27/28 loss by flow cytometry and suppression analysis (BrdU incorporation assay) to examine for the tolerogenic T cell phenotype.

## Results

The exosome-free complete RPMI control group and the tumor cell line conditioned growth medium without exosome group, showed CD3+CD8+ T cell’s CD27/28 loss to be at most 12% of the population. In contrast, CD27/28 loss, when CD3+CD8+ T cells were incubated with tumor conditioned growth medium and TD exosomes, were at the most 50% of the population. Congruent loss of CD27/28 expression was associated with an increased ability to suppress responder T cell proliferation, indicating immunosuppression.

## Conclusions

This research has identified TD exosomes from multiple cancer cell lines as a factor causing the CD3+CD8+ T cell tolerogenic phenotype. Furthermore, the lack of cell-to-cell contact needed between CD3+CD8+ T cells and the cancer cell lines to trigger this tolerogenic phenotype implies that CD3+CD8+ T cell exposure to the tumor microenvironment may not be required for these cells to become suppressor cells. Current research is involved in the purification of the factor(s) from the TD exosomes that may be causing this effect. Upon further identification of this factor(s) it could be used to better understand the etiology of the dysfunction in immunosurveillance due to immunosubversion.

